# Biochemical and Functional Characterization of LIZ, a Marine-Derived Serine Protease That Degrades Cross-Linked Fibrin and Attenuates Experimental Thrombosis

**DOI:** 10.3390/pharmaceutics18101294

**Published:** 2026-10-12

**Authors:** Sang-Gu Yun, Jung Eun Park

**Affiliations:** 1 Department of Biomedical Science, College of Natural Sciences, Chosun University, Jeonnam-Gwangju Integrated Special City 61452, Republic of Korea; 2 Department of Integrative Biological Science & BK21 FOUR Educational Research Group for Age-Associated Disorder Control Technology, Chosun University, Jeonnam-Gwangju Integrated Special City 61452, Republic of Korea

**Keywords:** fibrinolysis, serine protease, cross-linked fibrin, fibrinogenolysis, experimental thrombosis, marine enzyme, biopharmaceutical development

## Abstract

**Background/Objectives:** Direct fibrin-degrading proteases may provide a mechanistically distinct approach to clot proteolysis, but their development requires definition of catalytic behavior, fibrin selectivity, molecular identity, and safety. We characterized an activity-containing preparation from the marine polychaete *Lepidasthenia izukai*, designated LIZ. **Methods:** LIZ was obtained by ammonium sulfate fractionation and sequential chromatography. N-terminal sequencing, inhibitor profiling, chromogenic-substrate screening and S-2288 kinetics, fibrinogenolysis, direct fibrin-degradation assays, a κ-carrageenan rat-tail thrombosis model, and cell viability were evaluated. **Results:** The final preparation showed a predominant band at approximately 28 kDa. N-terminal sequencing yielded IMGGSEAS, providing sequence-level characterization without definitive molecular identification. PMSF abolished activity and DFP reduced it to 9.8%, supporting a serine-protease biochemical profile. For S-2288, *Km* was 0.029 ± 0.003 mM and apparent *kcat*/*Km* was 39.7 ± 4.3 mM^−1^ s^−1^. LIZ degraded fibrinogen Aα within 30 s and Bβ and γ within 20 min, decreased pre-formed fibrin-polymer turbidity, and degraded FXIIIa-cross-linked fibrin. In the pretreatment model, thrombotic tail length was 0.7 ± 1.0% with LIZ, 86.1 ± 3.2% with κ-carrageenan alone, and 0.5 ± 0.6% with uPA (*n* = 3/group). **Conclusions:** LIZ shows direct activity against structurally stabilized fibrin and proof-of-concept antithrombotic activity. However, the data do not establish definitive molecular identity, therapeutic thrombolysis, fibrin selectivity, relative potency, or a therapeutic safety window. Further molecular, pharmacological, and safety studies are required.

## 1. Introduction

Fibrin formation and fibrinolysis are tightly coordinated processes that preserve vascular integrity. Thrombin converts soluble fibrinogen into fibrin, whereas plasmin is the principal physiological enzyme responsible for fibrin degradation [1,2,3]. Persistent or excessive fibrin deposition contributes to intravascular thrombosis and remains an important pharmacological target [4,5]. Conventional fibrinolytic therapy relies largely on plasminogen activation, whereas proteases capable of directly degrading fibrin may provide a mechanistically distinct route to clot proteolysis [6,7].

For a newly isolated fibrinolytic protease to progress beyond biochemical characterization, several development-relevant questions must be addressed. These include whether the preparation acts directly on structurally mature fibrin, whether fibrin degradation can be distinguished from broader proteolysis of circulating coagulation proteins, and whether the active molecule can ultimately be identified, produced, formulated, and standardized with reproducible potency and stability [8,9]. These considerations are particularly important when evaluating a naturally derived enzyme as an early biopharmaceutical lead.

Marine organisms provide a diverse source of fibrinolytic enzymes with substantial variation in molecular mass, catalytic conditions, inhibitor sensitivity, substrate preference, and fibrin specificity [10,11,12,13,14]. Polychaetes are of particular interest because several fibrin(ogen)olytic serine proteases have been reported from this group [15,16,17], including three thermostable alkaline fibrinolytic serine proteases, CTSP1–3, from *Cirriformia tentaculata* [18]. These findings support continued investigation of polychaetes as sources of functionally distinct fibrinolytic proteases.

*Lepidasthenia izukai* is a marine polychaete reported from coastal waters of Republic of Korea, Japan, and China. A fibrin(ogen)olytic activity from *L. izukai* was previously described in a Master’s thesis [19], but has not been reported in a peer-reviewed journal. In the present study, we isolated an activity-containing preparation, designated LIZ, and characterized its purification behavior, serine-protease inhibitor profile, pH and temperature dependence, chromogenic-substrate profile, S-2288 kinetics, and cleavage of selected protein substrates. We further examined fibrinogen degradation, direct degradation of pre-formed and FXIIIa-cross-linked fibrin, and proof-of-concept antithrombotic activity in a κ-carrageenan-induced rat-tail pretreatment model. The aim was to define the biochemical and functional evidence supported by the current dataset while clearly identifying unresolved questions of molecular identity, selectivity, safety, and pharmaceutical development.

## 2. Materials and Methods

### 2.1. Materials and Collection of Lepidasthenia izukai

PMSF, DFP, TPCK, TLCK, EDTA, EGTA, 1,10-phenanthroline, DTT, prothrombin, fibrinogen, thrombin, plasmin, azocasein, collagen IV, γ-globulin, FXIIIa, and bovine serum albumin were obtained from Sigma (St. Louis, MO, USA). Chromogenic substrates were obtained from Chromogenix (Milan, Italy). HiPrep 16/10 Q FF, Mono Q 4.6/100 PE, Superdex 75 10/300 GL, and PD-10 columns were obtained from Amersham Pharmacia Biotech (Uppsala, Sweden). *L. izukai* specimens were collected from the coast of Wando Island, Republic of Korea (34°20′27″ N, 126°9′5″ E), washed immediately, frozen, and stored at −70 °C until use. Specimens were supplied by the laboratory of Prof. S. M. Yoon, Chosun University.

### 2.2. Purification and N-Terminal Sequencing of LIZ

**Figure 1:**
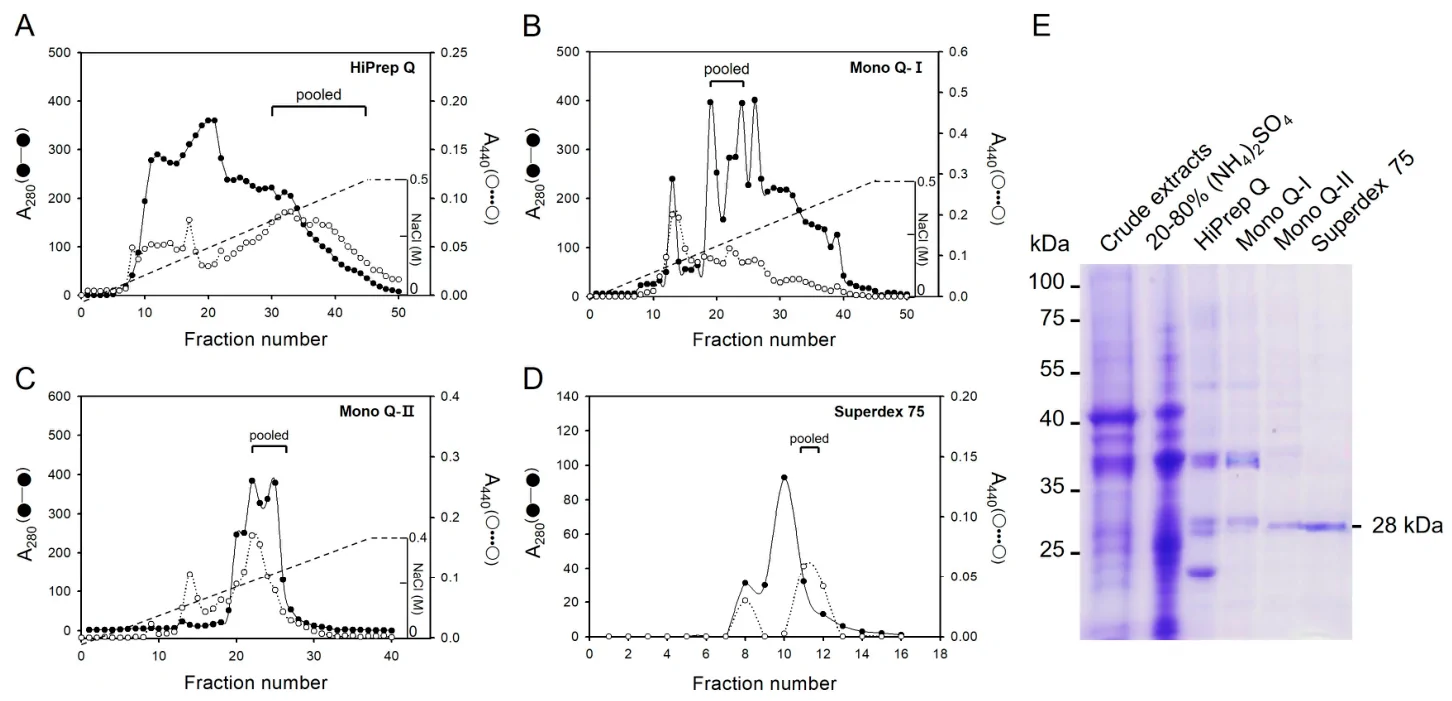
Activity-guided purification of the LIZ protease preparation from *Lepidasthenia izukai*. Sequential chromatographic purification of LIZ was performed using (**A**) HiPrep Q anion-exchange chromatography, (**B**) Mono Q-I anion-exchange chromatography, (**C**) Mono Q-II anion-exchange chromatography, and (**D**) Superdex 75 size-exclusion chromatography. Proteolytic activity (open symbols) and protein absorbance at 280 nm (filled symbols) were monitored in each fraction. Dashed lines in the anion-exchange chromatograms indicate the NaCl gradient. Fractions selected for continued activity-guided LIZ purification were pooled as indicated by the brackets and subjected to the subsequent purification step; the brackets do not necessarily denote the absolute maximum activity signal in each chromatogram. (**E**) SDS-PAGE analysis of samples obtained during the sequential purification of LIZ. The final Superdex 75 fraction showed a predominant protein band with an apparent molecular mass of approximately 28 kDa.

Frozen *L. izukai* tissue (approximately 50 g) was thawed in 100 mL ice-cold 25 mM Tris-HCl (pH 7.5), homogenized, and stirred for 6 h at 4 °C. After centrifugation at 12,000× *g* for 15 min at 4 °C, the supernatant was sequentially fractionated with 20% and 80% saturated ammonium sulfate. The 20–80% precipitate was collected, dissolved in 25 mM Tris-HCl (pH 7.5), and desalted on a PD-10 column. The sample was applied to a HiPrep 16/10 Q FF column equilibrated with 25 mM Tris-HCl (pH 7.5), and bound proteins were eluted with a 0–0.5 M NaCl gradient. Activity-containing fractions were pooled, desalted, and subjected to two sequential Mono Q 4.6/100 PE steps using 0–0.5 M and 0–0.4 M NaCl gradients, respectively. Fractions selected for continued purification were chosen based on the combined consideration of proteolytic activity and SDS-PAGE protein profiles. The tracked activity-containing fraction was then separated on a Superdex 75 10/300 GL column equilibrated with 25 mM Tris-HCl (pH 7.5) containing 0.15 M NaCl. The resulting activity-containing fraction was used as the final LIZ preparation. Protein concentration was determined by the Bradford method [20]. When more than one proteolytic-activity region was present in a chromatogram, the bracket in Figure 1 indicates the region selected for continuation of the LIZ purification sequence rather than necessarily the absolute maximum activity signal. The Superdex 75 step was used as an orthogonal size-exclusion polishing step after ion-exchange chromatography.

N-terminal amino acid sequencing of the final LIZ preparation was performed by Edman degradation as previously described for CTSP1–3 [18]. The sequence obtained for LIZ was compared with the reported N-terminal sequences of CTSP1, CTSP2, and CTSP3 from *Cirriformia tentaculata*.

### 2.3. Protease Activity Assay

Protease activity during purification was assessed using azocasein. The reaction mixture (200 μL) contained 0.5% azocasein, 25 mM Tris-HCl (pH 7.5), and 0.5 μg enzyme and was incubated at 37 °C for 30 min. The reaction was terminated by adding 100 μL of 10% trichloroacetic acid, followed by centrifugation at 10,000× *g* for 10 min. Absorbance of the TCA-soluble supernatant was measured at 440 nm. Protease activity was expressed in arbitrary activity units (AU) based on the absorbance change at 440 nm under the specified assay conditions using a SpectraMax 190 microplate reader (Molecular Devices, San Jose, CA, USA). No conversion of A440 values to micrograms of hydrolyzed azocasein was applied.

### 2.4. Chromogenic-Substrate Specificity and Coagulation-Related Protein Cleavage

Amidolytic activity was screened using S-2288, S-2238, S-2251, S-2366, S-2302, S-2444, S-2765, Boc-Val-Pro-Arg-*pNA* (VPR), and Boc-Leu-Gly-Arg-*pNA* (LGR). Reactions were performed in 100 μL containing 0.5 μg LIZ and 0.4 mM substrate for 1 h at 37 °C. Hydrolysis of the p-nitroanilide substrates was monitored at 405 nm, and blank-corrected endpoint A405 values were expressed relative to S-2288. This fixed-enzyme screening format was used to compare signals across substrates under identical conditions and was not intended to define catalytic efficiency. For S-2288 kinetic analysis, initial reaction rates were measured over a range of substrate concentrations and fitted to the Michaelis–Menten equation to obtain *Km* and *Vmax*. Apparent *kcat* was calculated from *Vmax* using an approximate molecular mass of 28 kDa, and apparent catalytic efficiency was expressed as *kcat*/*Km*. For qualitative protein-substrate cleavage, each substrate (10 μg) was incubated with LIZ (0.5 μg) for 10 min at 37 °C and analyzed by 12% SDS-PAGE. BSA, type IV collagen, and γ-globulin were included as non-coagulation proteins to provide a limited qualitative assessment of broader proteolysis; this panel was not designed to establish comprehensive protein selectivity.

### 2.5. Effects of pH, Temperature, Inhibitors, and Metal Ions

The apparent pH dependence of LIZ activity was examined for 1 h with S-2288 using 25 mM sodium acetate (pH 4.0–5.0), 25 mM PIPES (pH 5.5–6.0), 25 mM phosphate (pH 6.0–7.0), and 25 mM Tris-HCl (pH 7.5–11.0). Because different buffers were required to cover the pH range and a same-pH buffer-control matrix was not performed, the experiment is interpreted as apparent pH dependence under the specified buffer conditions; buffer-specific effects cannot be excluded. Temperature-dependent catalytic activity was measured by S-2288 hydrolysis for 1 h at the indicated temperatures. For inhibitor studies, LIZ (0.5 μg) was pre-incubated with each compound for 10 min at 37 °C before S-2288 hydrolysis was measured, and residual activity was expressed relative to the untreated control. TPCK and aprotinin were tested at 0.1 mM based on their established effective concentration ranges, whereas the other inhibitors and metal ions were tested at 1 mM.

### 2.6. Fibrinogenolytic and Fibrinolytic Assays

Fibrinogen degradation was assessed by incubating fibrinogen (100 μg) with LIZ (3 μg) in 25 mM Tris-HCl (pH 7.5) at 37 °C. Aliquots were collected at 0, 0.5, 1, 2, 3, 4, 5, 6, 10, and 20 min. Reactions were stopped with PMSF, and cleavage products were analyzed by SDS-PAGE. The enzyme-to-fibrinogen mass ratio was approximately 1:33 (approximate molar ratio ~1:3, using apparent molecular masses of 28 and ~340 kDa), a loading selected to visualize the time course of chain cleavage rather than to estimate kinetic or therapeutic potency. Direct fibrinolytic activity was evaluated separately using fibrin-plate and turbidity assays. For the fibrin-plate assay, LIZ (1 μg), plasmin (1 μg), or PBS was applied to 5 mm wells in fibrin-containing plates and incubated for 21 h at 37 °C; the LIZ/plasmin comparison was qualitative and mass-based rather than activity-matched. For turbidity measurements, pre-formed fibrin polymer was generated from 90 μL fibrinogen (1 mg/mL) and 10 μL thrombin (17.7 U/mL). LIZ (1 or 2 μg) or plasmin (1.5 μg) was then added, and the decrease in absorbance at 350 nm was monitored at 37 °C. The turbidity assay was performed in three independent experiments, and data are presented as mean ± SD.

FXIIIa-cross-linked fibrin was generated by incubating fibrinogen (20 μg), FXIIIa (0.002 U), thrombin (0.02 U), and CaCl_2_ (1 mM) in 25 mM Tris-HCl (pH 7.5) for 1 h at 25 °C. LIZ (0.5 μg) was then added for 30 min at 37 °C, and reaction products were analyzed by 8% SDS-PAGE. Cross-link formation was verified in the no-LIZ control by the appearance of high-molecular-weight α–α and γ–γ fibrin species. Loss of these species after LIZ exposure was used as evidence of degradation of stabilized fibrin. The relatively high LIZ loading was selected to provide a clearly detectable qualitative endpoint within 30 min and was not intended for kinetic or potency estimation.

### 2.7. κ-Carrageenan-Induced Rat-Tail Thrombosis Model

Male Sprague–Dawley (SD) rats (6 weeks old) were purchased from OrientBio (Seongnam, Republic of Korea). All animal procedures were conducted in accordance with protocols approved by the Chosun University Institutional Animal Care and Use Committee (IACUC) (Jeonnam-Gwangju Integrated Special City, Republic of Korea; approval no. CIACUC2018-A0037; approved 12 November 2018). During acclimation, the rats were housed in stainless-steel cages with noncontact bedding under controlled environmental conditions (22 ± 2 °C, 12 h light/dark cycle) and were provided Certified Rodent Diet 5002 (LabDiet, distributed by OrientBio, Seongnam, Republic of Korea) and tap water ad libitum.

For the thrombosis experiment, rats were anesthetized with 1.5% isoflurane in a mixture of nitrous oxide (70%) and oxygen (30%). Rats then received intravenous PBS, uPA (5.24 mg/kg), or LIZ (40 μg/kg), as indicated. The LIZ dose of 40 μg/kg was selected based on preliminary pilot experiments showing a measurable antithrombotic effect. Ten minutes later, the tail was ligated 10 cm proximal to the tip and κ-carrageenan (4 mg/kg) was administered intravenously to induce thrombosis [21]. The ligated tail was kept on ice for 10 min and then untied. For the quantitative analysis, animals were divided into the κ-carrageenan-only, κ-carrageenan + LIZ, and κ-carrageenan + uPA groups (*n* = 3 per group). In addition, untreated, PBS-only, and uPA-only groups (*n* = 3 per group) were included as photographic controls without κ-carrageenan challenge. Tails were photographed at the corresponding 24 h time point, and the visibly darkened thrombotic region was measured. A total of 18 animals were used in the in vivo study. Thrombotic tail length was calculated as the visibly darkened segment divided by the total visible tail length and expressed as a percentage. Individual animal values and mean ± SD were displayed graphically. A LIZ-only group without κ-carrageenan was not included in the original experiment.

### 2.8. SDS-PAGE

SDS-PAGE was performed using 8–12% polyacrylamide gels under reducing conditions [22]. Samples were mixed with reducing sample buffer, heated at 100 °C for 3 min, electrophoresed, and visualized with Coomassie Brilliant Blue.

### 2.9. Cell Viability Assay

Cell viability was assessed using the CellTiter 96^®^ AQueous Non-Radioactive Cell Proliferation Assay (Promega, Madison, WI, USA). RAW 264.7 macrophages (ATCC TIB-71, American Type Culture Collection, Manassas, VA, USA) were used as a preliminary immune-cell model for the initial cytotoxicity assessment of LIZ. RAW 264.7 cells were seeded in 96-well plates at a density of 5 × 10^4^ cells per well and allowed to adhere overnight. Cells were then treated with LIZ (0, 0.5, 1, 2, 3, and 5 μg/mL) for 24 h. This concentration range was selected independently for preliminary in vitro cytotoxicity screening and was not directly extrapolated from the 40 μg/kg in vivo dose, which was established based on preliminary pilot experiments. Following treatment, 20 μL of MTS reagent was added to each well, and the cells were incubated at 37 °C in 5% CO_2_ for 4 h. Absorbance was measured at 490 nm using a SpectraMax 190 microplate reader (Molecular Devices, San Jose, CA, USA). Cell viability was expressed relative to the untreated control.

### 2.10. Data Presentation and Statistical Considerations

Chromogenic-substrate and inhibitor/metal-ion assays were performed in three independent experiments and are presented as mean ± SD. For the in vitro biochemical assays, data were summarized descriptively as mean ± SD without inferential statistical comparisons because these experiments were intended for enzymatic characterization rather than prespecified hypothesis testing. The κ-carrageenan-challenged quantitative groups in the rat-tail experiment contained three independent animals each, and individual values together with mean ± SD. No prospective sample-size calculation or prespecified inferential statistical analysis was available for this proof-of-concept animal study; therefore, no retrospective *p*-values were introduced and the in vivo findings are interpreted descriptively. The experiment was not designed for dose-normalized or molar comparison of LIZ with uPA. Randomization and blinded thrombotic-tail-length measurement were not documented in the original study records. The fibrin-polymer turbidity assay was also performed in three independent experiments and is presented as mean ± SD.

## 3. Results

### 3.1. Purification Yield and Biochemical Evidence for a Serine Protease

Proteolytic activity from *L. izukai* extracts was enriched by 20–80% ammonium sulfate fractionation, HiPrep Q FF anion-exchange chromatography, two sequential Mono Q steps, and Superdex 75 size-exclusion chromatography. Specific activity increased from 207 to 1628 AU/mg, corresponding to an approximately 7.9-fold enrichment, while the recovered total activity decreased to 0.9% of the starting value (Table 1). The final fraction contained approximately 0.8 mg protein and showed a predominant band with an apparent molecular mass of approximately 28 kDa on reducing SDS-PAGE (Figure 1). This activity-containing fraction was designated the final LIZ preparation. The parallel increase in specific activity and enrichment of the ~28 kDa band supports co-enrichment of this band with the tracked proteolytic activity, but does not by itself demonstrate that all activity derives from a single molecular species. The marked loss of total activity reflects cumulative recovery loss during sequential purification; the Superdex 75 step provided only a modest increase in specific activity and functioned primarily as a polishing step.

Edman degradation of the final LIZ preparation yielded the N-terminal sequence IMGGSEAS. Over the eight residues examined, this sequence showed 75.0%, 62.5%, and 62.5% identity to CTSP1, CTSP2, and CTSP3 from *C. tentaculata*, respectively (Table 2). These data add sequence-level information to the biochemical characterization of LIZ, but the short N-terminal sequence is insufficient for definitive molecular identification.

LIZ activity was abolished by PMSF and reduced to 9.8% of control by DFP, whereas EDTA and 1,10-phenanthroline produced no inhibition (Table 3). Aprotinin reduced residual activity to approximately 45%. This pattern supports a serine-protease biochemical profile rather than a metalloprotease profile, but it does not establish molecular identity. The incomplete inhibition by aprotinin and the limited effects of TPCK and TLCK also do not justify assignment of LIZ to a narrower trypsin-like or chymotrypsin-like subclass. Most divalent cations had limited effects at 1 mM; Zn^2+^ and Ni^2+^ reduced residual activity to approximately 72% and 81%, respectively.

### 3.2. Catalytic Dependence on pH and Temperature

**Figure 2:**
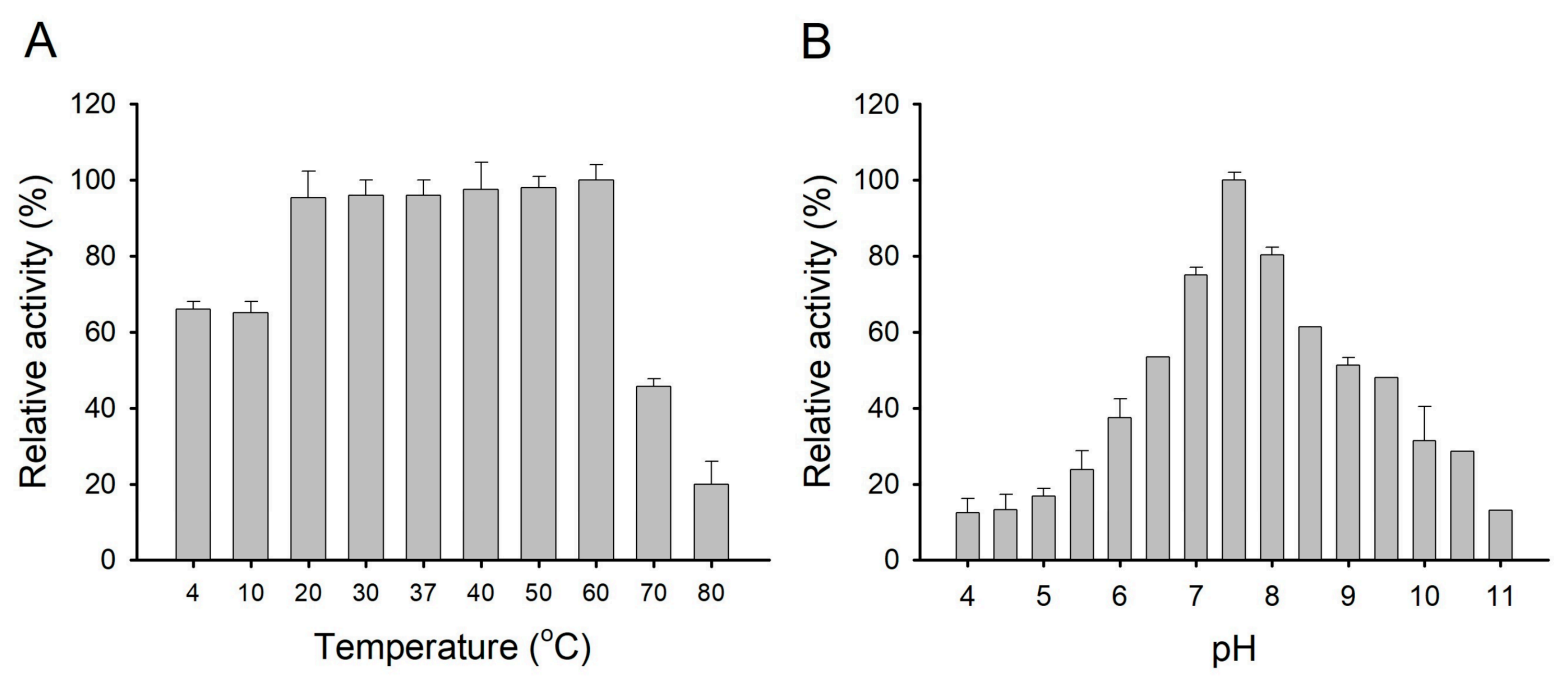
Effects of temperature and pH on LIZ activity. (**A**) Effect of temperature on LIZ activity. Enzymatic activity was measured at the indicated temperatures and expressed as relative activity, with the maximum activity observed at 60 °C defined as 100%. (**B**) Effect of pH on LIZ activity. Activity was determined over the indicated pH range and expressed relative to the maximum activity observed at pH 7.5, which was defined as 100%. Data are presented as mean ± SD.

Among the pH conditions tested, LIZ showed the highest S-2288-hydrolyzing activity at pH 7.5, with lower activity at more alkaline pH values above 8.5 (Figure 2B). Across the temperatures tested, catalytic activity was maintained over a broad range and reached its maximum at 60 °C (Figure 2A). Because activity was measured directly at each assay temperature rather than after a standardized pre-incubation period, these results describe temperature dependence of catalysis and should not be interpreted as a formal thermal-stability measurement.

### 3.3. Amidolytic Substrate Profile and Cleavage of Coagulation-Related Proteins

The chromogenic-substrate panel revealed a differentiated amidolytic profile (Table 4). S-2288 produced the highest signal and was defined as 100% relative activity; S-2238 and VPR showed approximately 51% and 53%, respectively, whereas S-2251 and S-2366 were weakly hydrolyzed. No increase above the corresponding substrate blank was detected for S-2302, S-2765, S-2444, or LGR under the screening conditions. These assay associations do not establish physiological identity as t-PA, thrombin, plasmin, or another coagulation protease. The contrast between VPR and LGR suggests sequence-context selectivity and is compatible with a contribution from P2 Pro versus Gly; however, because the substrates also differ at P3 (Val versus Leu), an independent P2 preference cannot be assigned from this pair alone. A formal analytical detection limit was not established, so 0% values denote no measurable signal above blank rather than absolute absence of hydrolysis.

Because S-2288 showed the highest activity in the initial screen, it was selected for kinetic analysis. Initial reaction rates obtained across a range of S-2288 concentrations were fitted to the Michaelis–Menten equation. The apparent *Km* was 0.029 ± 0.003 mM, *Vmax* was 2.46 ± 0.07 μmol min^−1^ mg^−1^, apparent *kcat* was 1.15 ± 0.032 s^−1^, and apparent *kcat*/*Km* was 39.7 ± 4.3 mM^−1^ s^−1^ (Table 5). These parameters quantitatively describe LIZ activity toward S-2288 but do not establish comprehensive substrate specificity because equivalent kinetic analyses were not performed for the other substrates.

**Figure 3:**
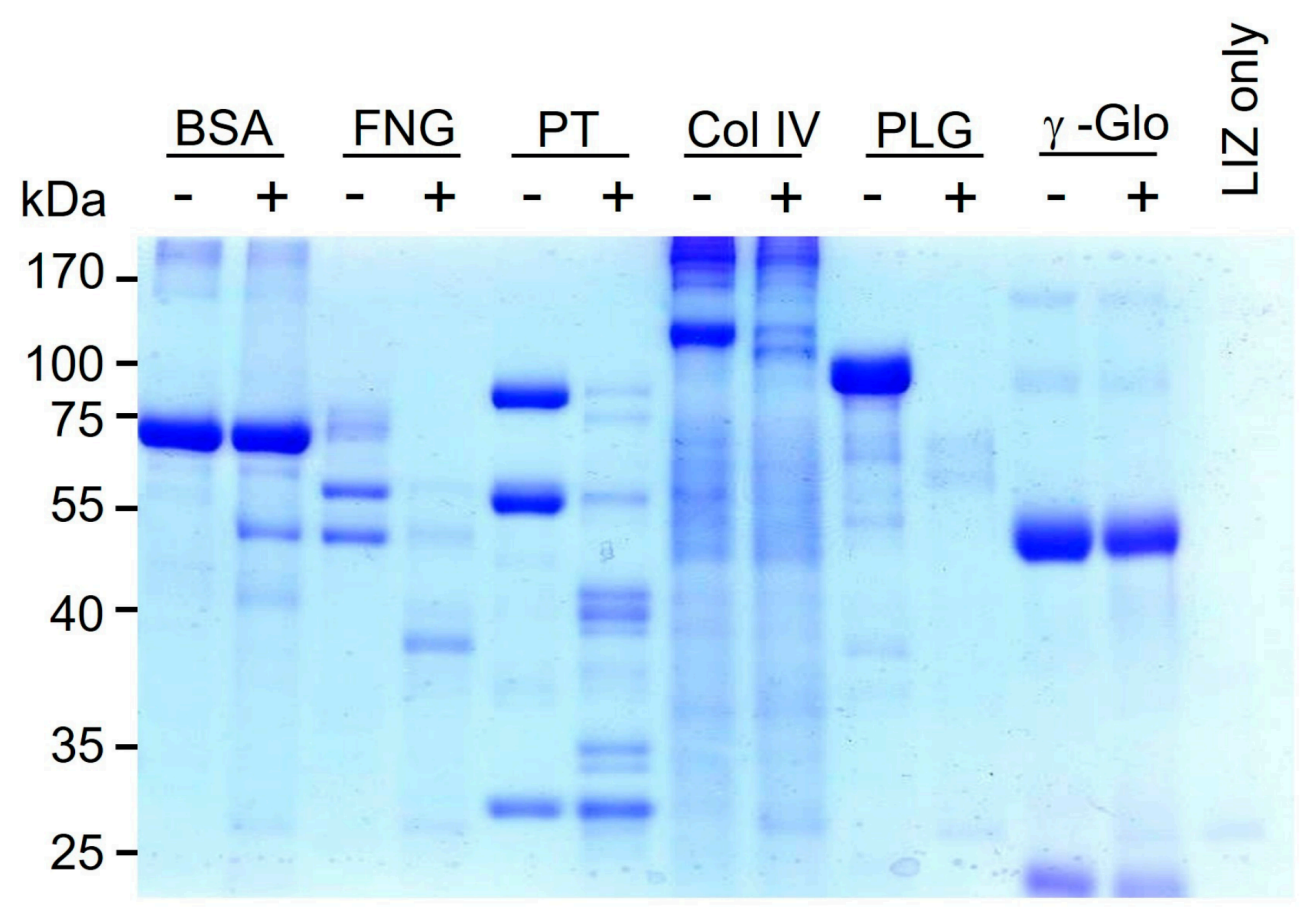
Proteolytic activity of LIZ toward selected protein substrates. Bovine serum albumin (BSA), fibrinogen (FNG), prothrombin (PT), type IV collagen (Col IV), plasminogen (PLG), and γ-globulin (γ-Glo) were incubated in the absence (−) or presence (+) of LIZ and analyzed by SDS-PAGE. The lane labeled “LIZ only” contains the final LIZ preparation alone. Changes in band intensity and the appearance of lower-molecular-weight fragments indicate substrate degradation by LIZ. Molecular mass markers are indicated on the left.

Incubation of the final LIZ preparation with selected proteins for 10 min produced clear fragmentation of fibrinogen, prothrombin, and plasminogen (Figure 3). SDS-PAGE demonstrates proteolytic cleavage but does not determine whether prothrombin or plasminogen is activated, inactivated, or converted into products with other biological functions. BSA and γ-globulin showed comparatively little change in their major bands. Type IV collagen showed a complex SDS-PAGE pattern even in the untreated control. After incubation with LIZ, modest changes in the band pattern were observed, consistent with limited proteolysis. These non-coagulation proteins are therefore interpreted only as a limited qualitative screen for broader proteolysis.

### 3.4. Time-Dependent Degradation of Fibrinogen

**Figure 4:**
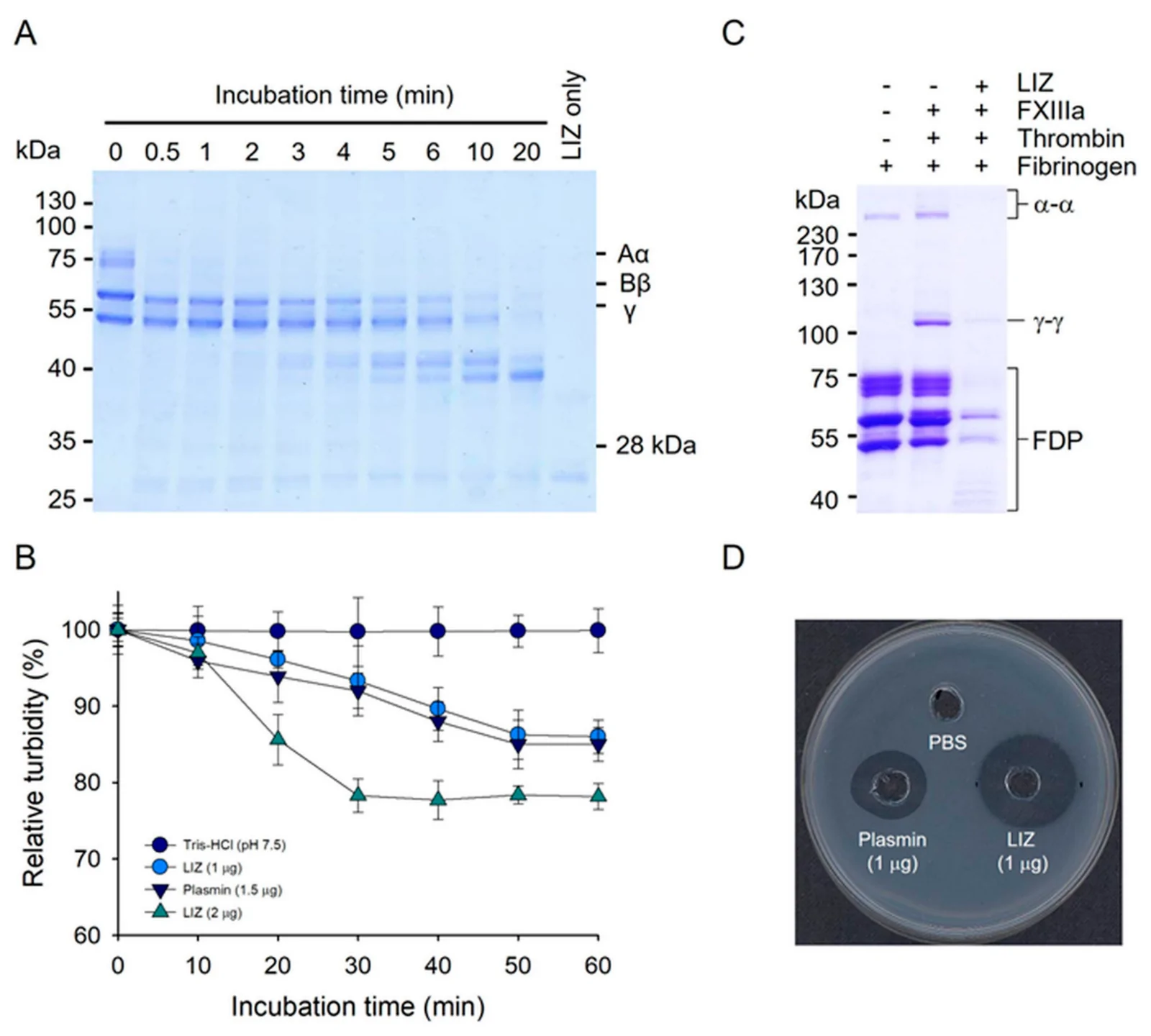
Fibrinogenolytic and fibrinolytic activities of LIZ. (**A**) Time-dependent degradation of fibrinogen by LIZ. Fibrinogen was incubated with LIZ for the indicated times and analyzed by SDS-PAGE. The positions of the fibrinogen Aα, Bβ, and γ chains and the final LIZ preparation (approximately 28 kDa) are indicated. (**B**) Degradation of pre-formed fibrin polymer by LIZ as assessed by turbidity. Relative turbidity was monitored over time in Tris-HCl buffer (pH 7.5) alone or in the presence of LIZ (1 or 2 μg) or plasmin (1.5 μg). Values are expressed relative to the initial turbidity. (**C**) Degradation of FXIIIa-cross-linked fibrin by LIZ. Fibrinogen was polymerized with thrombin in the presence of FXIIIa to generate stabilized fibrin and subsequently treated with LIZ. Samples were analyzed by SDS-PAGE. Cross-linked α–α and γ–γ fibrin species and fibrin degradation products (FDPs) are indicated. (**D**) Fibrinolytic activity of LIZ on a fibrin plate. LIZ (1 μg), plasmin (1 μg), or PBS was applied to the fibrin-containing plate, and fibrinolytic activity was visualized by the formation of clear lytic zones. Data in panel (**B**) are presented as mean ± SD from three independent experiments.

Under the specified high-enzyme-loading conditions, the fibrinogen Aα chain became undetectable within 30 s, whereas the Bβ and γ chains were progressively degraded and were no longer detectable by 20 min (Figure 4A). This pattern is consistent with Aα-initiated fibrinogenolysis followed by broader degradation of all three chains. Because no reference protease was included in this time-course experiment, the observed times are reported descriptively and are not interpreted as evidence of superior rate or potency.

### 3.5. Direct Activity Against Fibrin Polymer and FXIIIa-Cross-Linked Fibrin

Direct activity against fibrin was assessed independently of fibrinogenolysis using three complementary assays. Addition of LIZ to pre-formed fibrin polymer decreased turbidity at 350 nm over time (Figure 4B; mean ± SD from three independent experiments), and fibrin-plate treatment produced a visible lytic zone (Figure 4D). In the FXIIIa-cross-linked fibrin assay, the no-LIZ control contained high-molecular-weight α–α and γ–γ species, confirming formation of stabilized fibrin. These species were reduced after LIZ treatment and lower-molecular-weight products appeared (Figure 4C). Together, the data support direct proteolysis of fibrin, including FXIIIa-cross-linked fibrin. Because non-cross-linked and cross-linked fibrin were not compared quantitatively under identical conditions, the experiment does not establish preferential activity toward cross-linked fibrin.

### 3.6. Attenuation of Carrageenan-Induced Thrombosis in a Pretreatment Model

**Figure 5:**
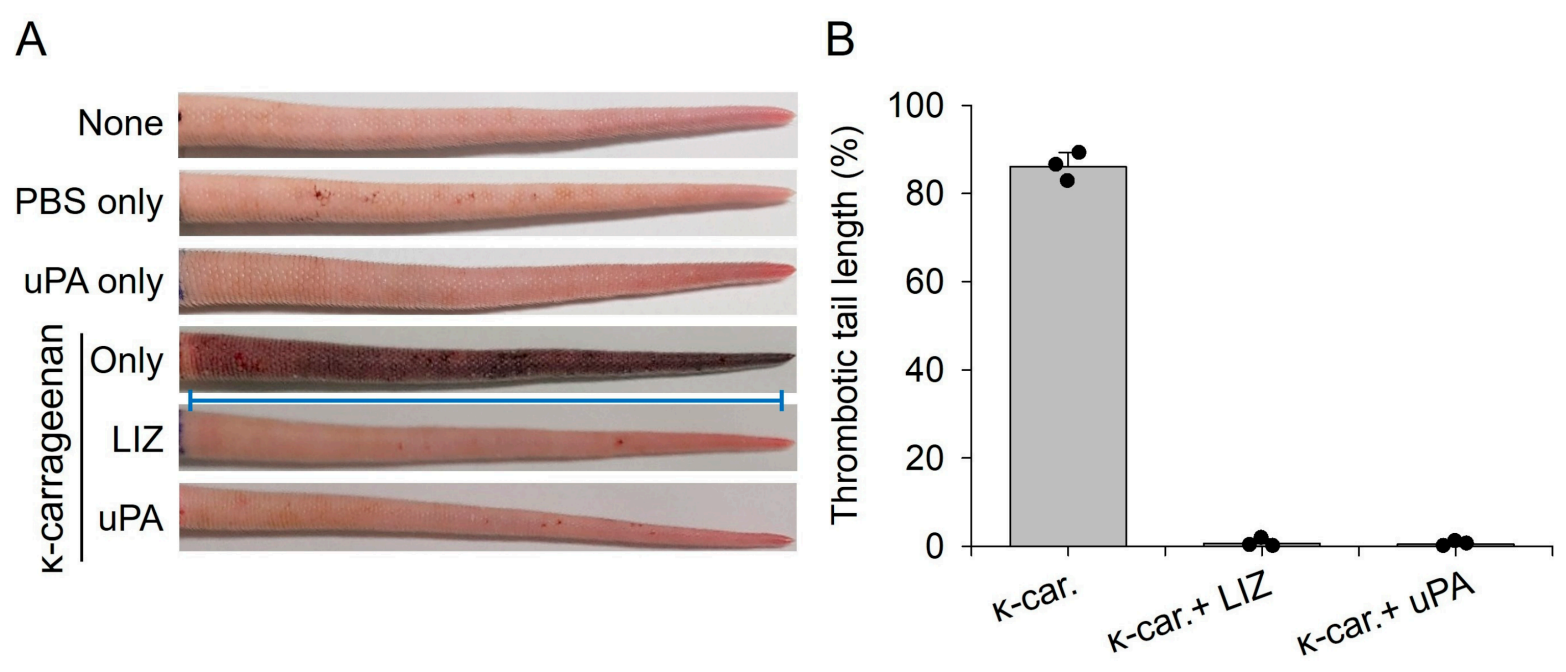
Effect of LIZ on κ-carrageenan-induced tail thrombosis in rats. (**A**) Representative photographs of rat tails 24 h after thrombosis induction. Rats received intravenous PBS, uPA (5.24 mg/kg), or LIZ (40 μg/kg), as indicated. Ten minutes after treatment, the tail was ligated and thrombosis was induced with κ-carrageenan (4 mg/kg). Untreated, PBS-only, and uPA-only animals are shown as controls without κ-carrageenan challenge. (**B**) Quantification of thrombotic tail length, calculated as the length of the visibly darkened thrombotic region divided by the total visible tail length × 100. Data are presented as mean ± SD (*n* = 3/group for the κ-carrageenan, κ-carrageenan + LIZ, and κ-carrageenan + uPA groups). Individual animal values are overlaid as points in panel (**B**). For measurement, the visibly darkened thrombotic region was defined as the segment extending from the proximal onset of visible discoloration to the tail tip. In panel (**A**), the blue bracket indicates the visibly darkened thrombotic region in the κ-carrageenan-only representative photograph.

LIZ was next evaluated in the κ-carrageenan-induced rat-tail model as a pretreatment proof-of-concept experiment. Representative tail photographs were obtained 24 h after challenge (Figure 5A). Thrombotic tail length was 86.1 ± 3.2% in the κ-carrageenan-only group, 0.7 ± 1.0% in the LIZ-pretreated group (40 μg/kg), and 0.5 ± 0.6% in the uPA-pretreated group (5.24 mg/kg; *n* = 3/group; Figure 5B). The result demonstrates an association between LIZ pretreatment and reduced thrombotic tail length under these experimental conditions. Because the groups were small and the LIZ and uPA doses were not activity-, mass-, or molar-matched, the experiment is interpreted descriptively and does not support a relative-potency comparison or demonstrate lysis of an established thrombus.

### 3.7. Initial Cytotoxicity Assessment of LIZ

As an initial in vitro safety-related assessment, RAW 264.7 cell viability was examined after 24 h exposure to LIZ. No appreciable reduction in viability was observed at concentrations up to 5 μg/mL under the conditions tested (Figure S1). This result is limited to the cell-viability assay and does not establish systemic safety, hemocompatibility, or a therapeutic safety window.

## 4. Discussion

The present study provides biochemical and proof-of-concept functional characterization of the LIZ preparation while also defining important limitations of the current evidence. Three findings are central. First, the inhibitor profile is consistent with serine-protease catalytic activity. Second, LIZ degrades soluble fibrinogen as well as pre-formed and FXIIIa-cross-linked fibrin, demonstrating activity across substrates of increasing structural complexity. Third, LIZ pretreatment was associated with a pronounced reduction in thrombotic tail length in the κ-carrageenan model. These findings support further characterization of LIZ as an early fibrinolytic lead, but they do not establish a molecularly defined drug substance, therapeutic thrombolysis, or pharmaceutical readiness.

The serine-protease interpretation is based primarily on complete inhibition by PMSF, strong inhibition by DFP, partial inhibition by aprotinin, and the absence of inhibition by EDTA or 1,10-phenanthroline (Table 3). However, inhibitor sensitivity alone cannot identify the active molecular species. Edman degradation yielded the N-terminal sequence IMGGSEAS, which shows partial similarity to CTSP1–3 from *C. tentaculata* (Table 2) and therefore adds sequence-level information. Nevertheless, the predominant ~28 kDa band, inhibitor profile, and eight-residue N-terminal sequence remain insufficient for definitive molecular identification. LC–MS/MS, peptide mapping, and/or gene-level identification will be required to establish sequence identity, support orthogonal purity testing, and enable recombinant production. The limited effects of TPCK and TLCK and incomplete inhibition by aprotinin also argue against assigning a narrower trypsin-like or chymotrypsin-like subclass from the current inhibitor panel.

Comparison with previously characterized fibrin(ogen)olytic proteases places LIZ within the broader group of polychaete and marine-invertebrate serine proteases while highlighting several distinguishing features (Table 6). Its apparent molecular mass (~28 kDa) is similar to NJF from *Neanthes japonica*, N-V protease from *Nereis (Neanthes) virens*, NJP from *N. japonica*, PLFP from *Periserrula leucophryna*, and CTSP1–3 from *Cirriformia tentaculata* [12,13,15,16,18]. In contrast to the more alkaline pH optima reported for NJF, NJP, and CTSP1–3, LIZ showed maximal activity near pH 7.5 [12,15,18]. N-V protease has a similar pH optimum (7.8) but a lower optimal temperature of 45 °C [13], whereas UFEII from *Urechis unicinctus* preferentially cleaves the fibrinogen γ chain before Bβ and Aα [14]. LIZ instead showed Aα-initiated fibrinogenolysis and direct degradation of fibrin polymer and FXIIIa-cross-linked fibrin. Because assay formats and conditions differ among studies, these comparisons are qualitative and do not establish relative catalytic potency.

The chromogenic-substrate data further support the need to distinguish screening behavior from physiological specificity. S-2288 was the most efficiently hydrolyzed substrate in the panel, and kinetic analysis provided quantitative parameters for this substrate (Table 5). However, S-2288 is not uniquely diagnostic of t-PA activity [23], and equivalent kinetic measurements were not obtained for the other substrates. Likewise, the VPR-versus-LGR contrast suggests sequence-context selectivity but cannot isolate a P2 preference because the two peptides also differ at P3. The cleavage of prothrombin and plasminogen is mechanistically important but remains functionally unresolved: SDS-PAGE demonstrates fragmentation, not whether these zymogens are activated or inactivated. Broader mapping of plasma-protein cleavage and functional assays of the resulting products will therefore be necessary to define both mechanism and off-target risk.

Fibrinogenolysis is relevant to clot proteolysis but also represents a potential selectivity liability [24]. Under the present high-enzyme-loading conditions, LIZ initiated cleavage at Aα and subsequently degraded Bβ and γ (Figure 4A). Because no reference protease was included in the time-course experiment, these observations should not be interpreted as a comparative measure of speed or potency. Degradation of circulating fibrinogen could reduce fibrin selectivity and increase bleeding risk [25]. Future development-oriented studies should therefore quantify a fibrin-versus-fibrinogen selectivity window using concentration-response analysis, plasma-based clot-lysis assays, fibrinogen-depletion measurements, and kinetic endpoints rather than relying on endpoint gel patterns alone.

The most distinctive functional finding is the direct activity of LIZ against structurally stabilized fibrin. Factor XIII-mediated cross-linking increases the mechanical and proteolytic resistance of fibrin [26,27,28]. In the present study, LIZ reduced cross-linked α–α and γ–γ species, while fibrin-polymer turbidity and fibrin-plate assays independently supported direct fibrin degradation (Figure 4B–D). These complementary assays strengthen the conclusion that LIZ can act on fibrin itself rather than only on soluble fibrinogen. However, the current experiments do not quantify catalytic efficiency against cross-linked fibrin, and matched non-cross-linked versus cross-linked substrates were not compared under identical conditions. Quantitative clot-lysis kinetics in plasma or whole-blood systems will be necessary to determine the pharmacological relevance of this activity.

The in vivo experiment must be interpreted within the limits of its pretreatment design. LIZ was administered before κ-carrageenan challenge, so the study addresses attenuation of thrombus formation rather than therapeutic dissolution of an established thrombus. The quantitative groups contained only three animals, no prospective sample-size calculation or prespecified inferential analysis was available, and randomization and blinding were not documented. In addition, the uPA and LIZ doses differed substantially and were not selected on the basis of matched proteolytic activity or molar exposure. Accordingly, the uPA group serves only as a qualitative positive-control reference and does not permit a potency comparison. Follow-up pharmacology should include treatment after thrombus establishment, adequately powered groups, prespecified randomization and blinded outcome assessment, dose–response analysis, and explicit bleeding and systemic-toxicity endpoints. A LIZ-only, non-carrageenan group should also be included to evaluate effects attributable to LIZ independently of the thrombosis challenge.

Safety and developability remain major unresolved issues. The RAW 264.7 assay showed no appreciable loss of cell viability up to 5 μg/mL, but this is only an initial in vitro observation. RAW 264.7 macrophages were selected as a preliminary immune-cell model because macrophages can respond sensitively to foreign proteins and bioactive materials; however, this assay was not intended to represent vascular-specific toxicity. Further evaluation using vascular endothelial and hematological cell types will be required. Bleeding, hemolysis, systemic toxicity, and immunogenicity were not established in the present study, and potential immunogenicity cannot be meaningfully assessed until the complete molecular sequence is known. From a pharmaceutics perspective, progression would require molecular identification and recombinant production, orthogonal purity and potency assays, mapping of fibrin and off-target cleavage sites, formulation and storage-stability studies, pharmacokinetic and biodistribution analysis, and comprehensive coagulation and safety evaluation. Because protein-therapeutic activity can be altered by aggregation, chemical degradation, excipients, and processing conditions [9], formulation and manufacturing controls will be essential. If systemic fibrin selectivity proves inadequate, localized or clot-targeted delivery could be considered in later-stage development.

Taken together, the strength of the study lies in the internally consistent demonstration that the LIZ preparation displays a serine-protease biochemical profile and degrades fibrin-related substrates at several levels of structural organization, including FXIIIa-cross-linked fibrin, with accompanying proof-of-concept antithrombotic activity in a pretreatment model. N-terminal sequencing and S-2288 kinetic analysis extend the molecular and catalytic characterization, while the cell-viability experiment provides a limited initial safety-related observation. The principal limitations are equally clear: definitive molecular identity has not been established; kinetic characterization is restricted to S-2288; fibrin selectivity and broader substrate specificity remain incompletely defined; the functional consequences of prothrombin and plasminogen cleavage are unknown; and the small prophylactic animal study does not demonstrate therapeutic thrombolysis or a therapeutic safety window. The current findings therefore support further molecular, biochemical, pharmacological, and safety characterization rather than a claim of established therapeutic efficacy.

## 5. Conclusions

The final LIZ preparation from the marine polychaete *L. izukai* contains a predominant protein band at approximately 28 kDa and exhibits an inhibitor profile consistent with serine-protease activity. It degrades all three fibrinogen chains and acts directly on pre-formed fibrin polymer and FXIIIa-cross-linked fibrin. In the κ-carrageenan pretreatment model, LIZ was associated with reduced thrombotic tail length, and N-terminal sequencing and S-2288 kinetic analysis provided additional sequence-level and catalytic characterization. However, the present data do not establish definitive molecular identity, broad substrate specificity, fibrin selectivity, lysis of established thrombi, relative potency versus uPA, or a therapeutic safety window. Further molecular identification, quantitative fibrin-versus-fibrinogen selectivity analysis, functional assessment of coagulation-protein cleavage, pharmacokinetic evaluation, and comprehensive studies of bleeding, hemolysis, systemic toxicity, and immunogenicity are required before the therapeutic potential of LIZ can be established.

## Figures and Tables

**Table 1:** Activity-guided purification of the LIZ preparation from *Lepidasthenia izukai*.

Purification Step	Total Protein (mg)	Total Activity (AU)	Specific Activity (AU/mg)	Yield (%)
Crude extract	700	145,038	207	100
20–80% (NH_4_)_2_SO_4_	450	104,798	232	72.3
HiPrep Q FF 16/10	19.2	20,086	1046.1	13.8
Mono Q-I 4.6/100 PE	3.7	5307	1434.3	3.7
Mono Q-II 4.6/100 PE	3.15	4797	1523.1	3.3
Superdex 75 10/300 GL	0.8	1302	1628	0.9

Protease activity is reported in arbitrary activity units (AU) based on the original A440-based activity calculation. No conversion of A440 to micrograms of hydrolyzed azocasein was applied.

**Table 2:** Comparison of the N-terminal amino acid sequence of LIZ with fibrinolytic serine proteases from *Cirriformia tentaculata*.

Protease	Source	N-Terminal Sequence	Identity with LIZ (%)
LIZ	*L. izukai*	IMGGSEAS	100
CTSP 1	*C. tentaculata*	IMNGSPAS	75.0
CTSP 2	*C. tentaculata*	IMYGQEAA	62.5
CTSP 3	*C. tentaculata*	IIGGTEAD	62.5

Sequence identity was calculated over the eight N-terminal residues determined for LIZ. CTSP1, CTSP2, and CTSP3 sequences were obtained from ref. [18].

**Table 3:** Effects of protease inhibitors and metal ions on LIZ activity.

Additive	Concentration (mM)	Relative Activity (%)
Control	—	100 ± 3.2
TPCK	0.1	68 ± 4.3
TLCK	1	103 ± 9.2
PMSF	1	0 ± 0
Aprotinin	0.1	45 ± 0.3
EGTA	1	109 ± 6.2
EDTA	1	111 ± 2.2
DTT	1	102 ± 0.4
DFP	1	9.8 ± 1.1
1,10-Phenanthroline	1	105 ± 0.55
Ca^2+^	1	100 ± 1.3
Cu^2+^	1	103 ± 6.4
Mg^2+^	1	101 ± 5.9
Mn^2+^	1	94 ± 1.3
Ni^2+^	1	81 ± 1.5
Zn^2+^	1	72 ± 5.2

S-2288 was used as the substrate. Values are those reported in the original dataset from three independent experiments (mean ± SD).

**Table 4:** Amidolytic activity of LIZ against synthetic protease substrates.

Substrate	Common Assay Association	Relative Activity * (%)
S-2288	t-PA	100 ± 5
S-2238	Thrombin	51 ± 11
VPR	Thrombin	53 ± 1.2
S-2251	Plasmin	5.5 ± 1.2
S-2366	FXIa	4.3 ± 0.2
S-2302	FXIIa	0 ± 0
S-2765	FXa	0 ± 0
S-2444	Urokinase	0 ± 0
LGR	FXa	0 ± 0

* Relative activity was normalized to S-2288. Values are those reported in the original dataset from three independent experiments (mean ± SD). The “common assay association” designation reflects conventional analytical use of each synthetic substrate and does not assign physiological enzyme identity or in vivo substrate specificity to LIZ. Values of 0% indicate no measurable signal above the corresponding substrate blank under these assay conditions; a formal analytical limit of detection was not established.

**Table 5:** Kinetic parameters of LIZ for hydrolysis of the chromogenic substrate S-2288.

Enzyme	Substrate	*Km* (mM)	*Vmax*	Apparent *kcat* (s^−1^)	Apparent *kcat*/*Km* (mM^−1^ s^−1^)
LIZ	S-2288	0.029 ± 0.003	2.46 ± 0.07	1.15 ± 0.032	39.7 ± 4.3

*Km* and *Vmax* were determined by Michaelis–Menten analysis of S-2288 hydrolysis. Apparent *kcat* was calculated using an apparent molecular mass of approximately 28 kDa for LIZ, and apparent *kcat*/*Km* represents catalytic efficiency. Because the active molecular species has not been definitively identified or quantified independently within the preparation, *kcat* and *kcat*/*Km* should be regarded as apparent values. Values are presented as mean ± SD from three independent experiments.

**Table 6:** Comparison of the biochemical and functional properties of LIZ with representative fibrin(ogen)olytic proteases from polychaetes and related marine invertebrates.

Protease	Source	MW (kDa)	Optimal pH	Optimal Temperature	Inhibitor/Protease Profile	Fibrinogen Cleavage	Major Fibrinolytic Feature	Ref.
LIZ	*L. izukai*	~28	7.5	60 °C	PMSF, DFP-sensitive serine protease	Aα first; Bβ and γ subsequently	Fibrin polymer and FXIIIa-cross-linked fibrin degradation	This study
NJF	*N. japonica*	28–32	9.0	60 °C	PMSF-sensitive serine protease	Aα → Bβ → γ	Fibrin(ogen)olytic activity	[12]
N-V protease	*N. virens*	29	7.8	45 °C	PMSF/DFP-sensitive serine protease	Aα > Bβ > γ	Direct fibrinolytic activity	[13]
UFEII	*U. unicinctus*	26.7	8.0	50 °C	Chymotrypsin-like serine protease	γ → Bβ → Aα	Direct fibrin degradation and plasminogen activation	[14]
NJP	*N. japonica*	28.6–33.5	9.0	40 °C	PMSF-sensitive serine protease	Aα → Bβ → γ	Thrombin-like fibrinolytic activity	[15]
PLFP	*P. leucophryna*	30	NR	NR	Plasmin-like serine protease	—	Direct artificial fibrin degradation; Lys-P1 preference	[16]
CTSP1–3	*C. tentaculata*	28.4–30.9	8.5–9.0	50–60 °C	PMSF/DFP-sensitive serine proteases	All chains degraded within 20 min	Fibrin polymer, cross-linked fibrin, and plasma clot degradation	[18]

NR, not reported in the cited study or not included in the present comparison. Molecular mass and optimal catalytic conditions are presented as reported in the corresponding references. Because assay conditions differed among studies, these values are intended for qualitative comparison and should not be interpreted as direct measurements of relative catalytic potency.

## Data Availability

The data presented in this study are available on request from the corresponding author. The data supporting the findings of this study are available from the corresponding author upon reasonable request. The data have not been deposited in a public repository because they consist primarily of experimental laboratory records and raw biochemical measurements that require contextual interpretation.

## Supplementary Materials

The following supporting information can be downloaded at https://www.mdpi.com/article/10.3390/pharmaceutics18101294/s1, Figure S1. Effect of LIZ on RAW 264.7 cell viability. RAW 264.7 cells were treated with LIZ at the indicated concentrations (0–5 g/mL) for 24 h. Cell viability was determined using the CellTiter 96^®^ AQueous Non-Radioactive Cell Proliferation Assay. Cell viability was expressed as a percentage relative to the untreated control. Data are presented as mean + SD from three independent experiments.

## Abbreviations

The following abbreviations are used in this manuscript:

BSA	bovine serum albumin
DFP	diisopropyl fluorophosphate
DTT	dithiothreitol
EDTA	ethylenediaminetetraacetic acid
EGTA	ethylene glycol-bis(β-aminoethyl ether)-N,N,N′,N′-tetraacetic acid
FDPs	fibrin degradation products
FNG	fibrinogen
FXIIIa	activated coagulation factor XIII
IACUC	Institutional Animal Care and Use Committee
LGR	Boc-Leu-Gly-Arg-p-nitroanilide
LIZ	Lepidasthenia izukai-derived protease preparation
PBS	phosphate-buffered saline
PIPES	piperazine-N,N′-bis(2-ethanesulfonic acid)
PLG	plasminogen
PMSF	phenylmethylsulfonyl fluoride
PT	prothrombin
SDS-PAGE	sodium dodecyl sulfate–polyacrylamide gel electrophoresis
t-PA	tissue-type plasminogen activator
TLCK	Nα-tosyl-l-lysine chloromethyl ketone
TPCK	N-tosyl-l-phenylalanine chloromethyl ketone
uPA	urokinase-type plasminogen activator
VPR	Boc-Val-Pro-Arg-p-nitroanilide
